# Age at infection as a key predictor of cyst burden in pigs experimentally infected with *Taenia solium*

**DOI:** 10.1186/s13071-025-06844-6

**Published:** 2025-09-24

**Authors:** Eloy Gonzales-Gustavson, Francesco Pizzitutti, Gabrielle Bonnet, Miguel Muro, Mayra Elizalde, Claudio Muro, Ricardo Gamboa, Gianfranco Arroyo, Sarah Gabriël, William K. Pan, Héctor H. Garcia, Seth O’Neal

**Affiliations:** 1 Department of Animal and Public Health, School of Veterinary Medicine, Universidad Nacional Mayor de San Marcos, Lima, Peru; 2 Geography Institute, Universidad San Francisco de Quito, Quito, Ecuador; 3 Centre for the Mathematical Modeling of Infectious Diseases, London School of Hygiene and Tropical Medicine, London, UK; 4 Center of Global Health, Universidad Peruana Cayetano Heredia, Lima, Peru; 5 Department of Translational Physiology, Infectiology and Public Health, Ghent University, Ghent, Belgium; 6 Nicholas School of Environment and Duke Global Health Institute, Duke University, Durham, NC USA; 7 School of Public Health, Oregon Health & Science University and Portland State University, Portland, USA

**Keywords:** *Taenia solium*, Pig cysticercosis, Age at infection, Innate immunity, Susceptibility at infection

## Abstract

**Background:**

*Taenia solium* cysticercosis is a zoonotic parasitic disease with significant public health implications, particularly in endemic regions of low- and middle-income countries. In pigs, cyst burden varies widely, with most harboring fewer than 10 cysts and only a small fraction carrying high cyst loads. Age has been identified as a key factor influencing infection susceptibility. However, inconsistencies in previous studies have hindered clear characterization of infection patterns and immunity. In this study, we conducted controlled experiments involving the infection of pigs with *T. solium* eggs to evaluate the relationship between pig age and susceptibility to infection.

**Methods:**

A total of 52 pigs from northern Peru, aged 4 to 22 weeks, were experimentally infected with *T. solium* eggs to examine age-related differences in cyst burden. Pigs were housed individually under controlled conditions and fed commercial pig diets. Infections were administered using an esophageal catheter, delivering 20,000 *T. solium* eggs in gelatin capsules. Six age groups were studied using a standardized egg pool to ensure consistency across infection rounds. After 10 weeks, necropsies were performed to count cysts in all muscles, the brain, and other organs. Weekly serological tests monitored seroconversion. Statistical models were used to analyze cyst counts and assess the effects of age and other predictors.

**Results:**

The number of live, degenerated, and total cysts was overdispersed, making a negative binomial model the most suitable choice to represent the data and their dependence on age at infection. Younger pigs showed low median live cyst count, similar to older pigs, while median cyst burden increased in pigs infected at intermediate ages, around natural weaning age. The negative binomial regression showed that age and a covariate inversely related to age at infection were significantly associated with cyst count at necropsy. Other covariates such as egg pool viability and sex did not significantly affect model performance. Serological tests confirmed seroconversion in all pigs.

**Conclusions:**

Our results show that younger pigs display partial protection against the development of cysticerci compared to those infected at the natural weaning age (around 9 to 12 weeks of age). Additionally, infection susceptibility then decreases with age in a way that is consistent with previous literature.

**Graphical Abstract:**

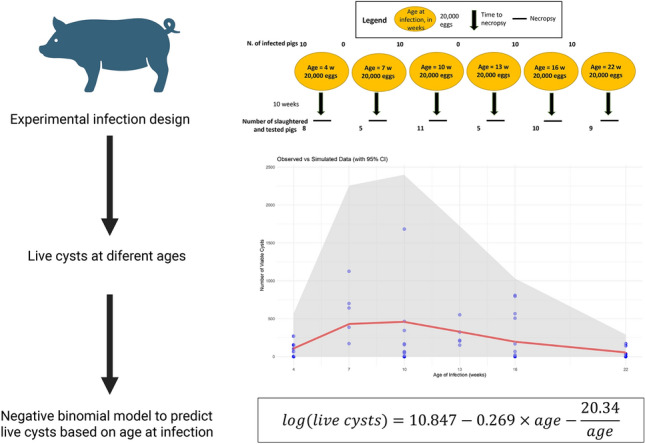

**Supplementary Information:**

The online version contains supplementary material available at 10.1186/s13071-025-06844-6.

## Background

*Taenia solium* cysticercosis is a zoonotic parasitic infection that poses significant public health challenges, particularly in low- and middle-income countries [1]. The parasite’s life cycle involves humans as the definitive host, with the adult tapeworm residing in the intestine, where it sheds proglottids and eggs in the host’s feces. When these eggs are ingested by pigs, they develop into the larval stage, known as cysticerci. Humans can also inadvertently ingest the eggs, leading to the development of cysticercosis in various tissues—most notably in the central nervous system, causing neurocysticercosis, a severe neurological disease [2].

In endemic regions, an estimated 1–3% of the human population harbors the adult *T. solium* tapeworm [3, 4], serving as the primary source of cysticercosis and leading to a pig infection prevalence of approximately 10–30% [5–7]. Pigs with high cyst burdens—hundreds to thousands of cysts, readily visible in the carcass during slaughter—represent only a small minority of infections in the population. The majority of infected pigs harbor fewer than 10 cysts [8] and are less likely to be detected during slaughter. Typically, more than 80% of the parasites are harbored by fewer than 20% of the hosts [9]. This phenomenon, known as parasite aggregation, is broadly attributed to several density-dependent mechanisms, both intrinsic and extrinsic to the pig, which contribute to the observed overdispersion in infection patterns [10]. Innate and acquired immunity are two such mechanisms that are known to play a critical role in limiting the number of *T. solium* cysts in pig populations [11].

Past evidence suggests that age at infection, sex, and the genetic strain of the host animal are potential contributors to variability in infection among various taeniid cestodes, with age emerging as the most influential factor [12, 13]. Age has been identified as contributing to innate resistance among naïve or previously unexposed animals challenged with different tapeworm species such as *Taenia taeniaeformis* in rats, *T. pisiformis* in rabbits, *T. saginata* in cattle, and *T. hydatigena* in sheep [13]. It has further been suggested that immunity may not fully prevent the establishment of new cysts but likely modulates the infection’s progression over time [11]. A few studies have utilized controlled exposure to more accurately characterize resulting infection intensity at different ages [14]. However, methodological inconsistencies—such as insufficient standardization of the infective dose, differences in egg batches, pig breeds, incomplete organ examinations, and the inability to detect cysts due to an inappropriately short time interval between infection and necropsy [12, 13]—weaken the strength of the evidence.

This significant variability in cyst burden presents challenges for implementing effective strategies for monitoring and evaluating the impact of potential control interventions [15, 16]. While understanding pig immunity is critical to determining what control or elimination interventions are likely to be most effective, the evidence in this regard remains limited and insufficiently precise to inform intervention modeling, even when considering studies with other taeniid cestodes.

The objective of this study was to accurately characterize differences in infection burden across age groups after a single infection, to gain a better understanding of age-related and innate immunity, as well as to inform representation of pig immunity in transmission simulation models designed to guide interventions. We achieved this through a controlled experiment designed to minimize as many sources of variation as possible, allowing us to determine differences in cyst burden in pigs infected with *T. solium* at varying ages.

## Methods

### Animals

The initial study design included 40 pigs, divided into four age groups: 4, 10, 16, and 22 weeks of age (10 per age group). Due to space limitations, infections were conducted in six separate rounds. After the fourth round, interim results suggested that more data were needed to improve precision around 10 weeks of age. Therefore, we added 12 more pigs: we created two new age groups (7 and 13 weeks, with five pigs each) and included two additional 10-week-old pigs. This brought the final total to 52 pigs. The pigs used in this study were purchased from nine different commercial farms in northern Peru, selected based on the availability of pigs of the required ages for each experimental round. The acquired pigs were of mixed breeds commonly found in the region. While mixed-breed pigs are more genetically variable than pure breeds, they are more representative of the pigs that are used in pig farming, hence ensuring better generalizability in our results. Five pigs were excluded—four died prior to the scheduled necropsy, and one had an aberrant serological response at baseline—leaving an analytical sample of 47 pigs (Table 1).

**Table 1:** Median, minimum, and maximum numbers of live, degenerated, and total cysts by age at infection, along with the corresponding number of pigs per group

Age at infection	Number of pigs	Live cysts			Degenerated cysts			Total cysts		
		Median	Minimum	Maximum	Median	Minimum	Maximum	Median	Minimum	Maximum
4 weeks	7	103	0	270	63	5	490	192	71	514
7 weeks	5	642	172	1126	17	3	25	645	180	1143
10 weeks	11	66	0	1682	12	0	326	172	1	1705
13 weeks	5	216	152	552	10	5	15	231	165	557
16 weeks	10	118.5	1	809	58.5	1	260	286	30	833
22 weeks	9	7	0	171	71	1	217	75	2	394

The pigs were housed in individual pens at the specific-pathogen-free facilities of the Center for Global Health at Cayetano Heredia University (UPCH) in Tumbes. The animals were fed exclusively on properly packaged commercial feed, stored at our facilities to prevent contamination. Water was provided ad libitum.

### Serological test

All pigs underwent serological screening to rule out prior exposure to *T. solium* before purchase, using lentil lectin-bound glycoprotein enzyme-linked immunoelectrotransfer blot assay (LLGP-EITB) [17] and antigen enzyme-linked immunosorbent assay (ELISA) (TsW8/TsW5 monoclonal antibody [mAb] set) [18]. All pigs were required to be negative on both tests (absence of any of the seven reactive bands on LLGP-EITB; optical density ratio < 1 on antigen ELISA) to be included in the study. Positive and negative control sera were included in each test run and were provided by the Cysticercosis Working Group in Peru. The mothers of all included pigs, except three due to owner refusal, were also screened to ensure the negative status of the sow. Throughout the duration of the experiment, weekly blood samples were also taken to monitor the evolution of seroconversion with both tests. These results will feed into a separate paper.

### Preparation of egg pools

Tapeworms for this experiment were sourced from control interventions in which human stool was collected from the community setting and screened for *T. solium* taeniasis using a combination of microscopy, coproantigen ELISA, and molecular confirmation of species using polymerase chain reaction (PCR) [19]. Stool was collected during bowel preparation prior to antiparasitic treatment. Gravid proglottids were collected and stored in saline solution at 4 °C for a maximum of 2 weeks, ensuring that eggs used in the experiment had not been exposed to antiparasitic drugs or preservatives. To minimize variability between infections due to differences in egg viability, we pooled *T. solium* eggs from multiple tapeworms for each round of infections, using a new pool for each round. The number of tapeworms used to create each pool was 10, 3, 7, 8, 6, 10, and 3, respectively. The number of tapeworms per round varied depending on tapeworm availability and the number of viable eggs available for each tapeworm; however, we managed to combine at least three tapeworms to prepare the pool used in each round. Each tapeworm originated from a different human donor from northwestern Peru (Piura and Tumbes regions).

Pools were prepared by mixing eggs harvested from gravid proglottids obtained from 3–10 different tapeworms. Egg viability (Evans blue stain) and percentage of activated oncospheres using the enzyme method and movement of the hexacanth embryo [20] were assessed for each proglottid and for the entire pool. The mean percentage of activated oncospheres across all pools was 82% (range 63–97%).

### Experimental design

**Fig. 1:**
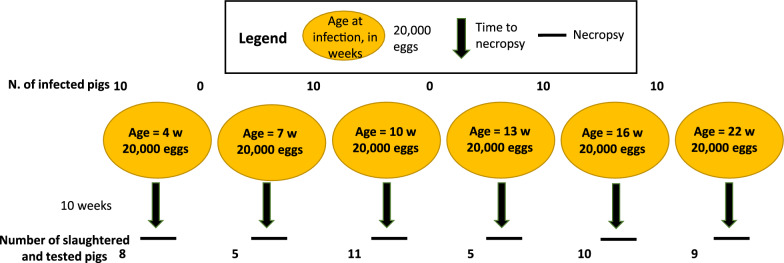
Schematic representation of the experimental design for the pig study: pigs of different ages at infection, indicated by the circles, were experimentally infected with 20,000 *T. solium* eggs to assess infection patterns and overdispersion across age at infection groups

In total, six different groups of pigs, corresponding to infections at 4, 7, 10, 13, 16, and 22 weeks of age, were experimentally infected over six rounds (Fig. 1). The same pool of eggs was used for each round, and pigs of different age groups were infected with eggs from the same pool. Infections were administered using an esophageal catheter, following a previously described method with slight modifications [21]. Briefly, each pig received 20,000 *T. solium* eggs suspended in olive oil in gelatin capsules, which were delivered through an esophageal catheter. The capsules were then flushed into the stomach using 20–100 m of bottled drinking water. All pigs were anesthetized with a combined intramuscular dose of ketamine (20 mg/kg) and xylazine (2 mg/kg). After the infection procedure, the pigs were monitored by a veterinary team for stress reaction (vital signs) and for signs of emesis or regurgitation of the capsules. Pigs were then maintained in individual pens for a period of 10 weeks until necropsy. All pigs were weighed at the time of infection, and the median, minimum, and maximum weight per group are provided as Supplementary Information in Table S1. For necropsy, pigs were induced to anesthesia with intramuscular ketamine (20 mg/kg) and xylazine (2 mg/kg) and then euthanized by intravenous sodium pentobarbital (100 mg/kg). The carcass was then dissected by trained staff using 3- to 5-mm slices of all muscles, brain, and other organs, and visually inspected to identify and count cysts, recording the number and type (viable or degenerated) of cysts for each animal.

### Data analysis

We used Kruskal–Wallis as a preliminary test to evaluate differences in live cyst counts between pigs infected at different ages. This nonparametric test was chosen due to its robustness against violations of normality assumptions and its ability to handle skewed data distributions commonly observed in cyst count outcomes. Significant differences detected by the Kruskal–Wallis test indicated the need for further exploration of age-related effects through more advanced modeling techniques.

We evaluated several statistical regression models, including Poisson, negative binomial, zero-inflated Poisson, zero-inflated negative binomial, and negative binomial family generalized estimating equations, for their ability to fit the observed counts of live *T. solium* cysts in pigs infected at different ages. Each model was assessed for its ability to predict the number of live cysts while accounting for the distribution of the count data and potential overdispersion. Several predictors were considered and incorporated into the models if they improved their fit to the data. These predictors included the farm of origin of the pigs, the round of infection (to account for potential variation between infection periods), the viability of each egg pool (to adjust for differences in the proportion of viable oncospheres across rounds), the inverse of age and quadratic age terms (to capture potential nonlinear effects of age on cyst counts), and the sex of the pigs. Model fit was evaluated using criteria such as the Akaike information criterion (AIC) and Bayesian information criterion (BIC), and the best-fitting model was selected based on these indices and residual diagnostics. Most of the analyses were performed using R [22] and the packages MASS [23], pscl [24], and ggplot2 [25], except for the generalized estimating equations, which were developed with the xtgee command in STATA [26].

## Results

The median number of live, degenerated, and total cysts, along with their respective ranges across different ages at infection, are presented in Table 1. A significant variation in the median number of live cysts was observed between age groups (Kruskal–Wallis *H*-test, *H* = 15.65, *df* = 5, *P* = 0.008), suggesting that the age at infection influences cyst burden in pigs. Specifically, younger pigs showed similarly low median cyst counts relative to the older ones, with a notable increase in cyst burden observed in pigs infected at intermediate ages. This variation is detailed in Table 1. These results suggest that age plays an important role in cyst development following egg ingestion.

The pattern observed for degenerated cysts was the inverse of that seen for live cysts (Table 1). The number of degenerated cysts was higher in younger and older pigs compared to those infected at intermediate ages, showing marginally significant differences between age groups (Kruskal–Wallis *H*-test, *H* = 10.29, *df* = 5, *P* = 0.07). The total number of cysts (live and degenerated combined) followed a trend comparable to that of live cysts but with less pronounced differences with age. These differences were marginally significant (Kruskal–Wallis *H*-test, *H* = 10.31, *df* = 5, *P* = 0.07). Median values for each group are provided in Table 1.

Only four pigs harbored cysts in the brain, with a total of seven cysts found in just two groups: pigs infected at 7 and 16 weeks of age. Each pig harbored one or two cysts, and all cysts were viable.

### Statistical model prediction

**Fig. 2:**
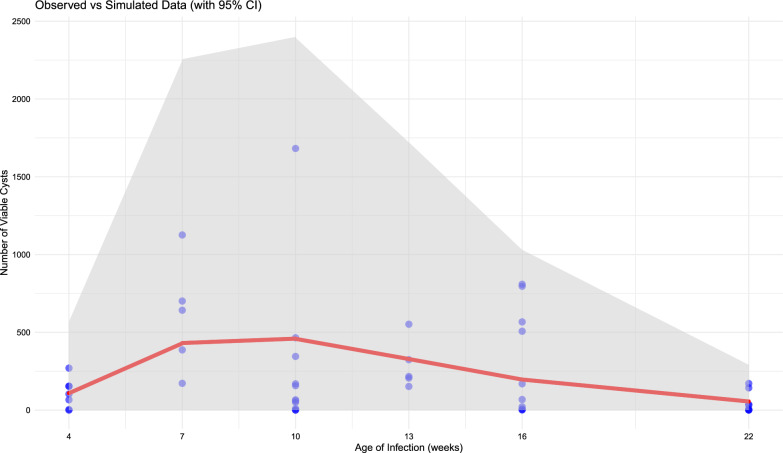
Number of live cysts (*y* axis) in pigs infected at different ages (*x* axis). Blue dots represent individual cyst counts, while the red line illustrates the predicted values from the negative binomial regression model, showing differences across ages. The shaded area represents the 95% credible intervals from the model, indicating the range of expected values

All statistical models yielded similar results. However, the negative binomial regression emerged as the most suitable model to predict the number of live cysts as a response variable based on age and other potential covariates. This conclusion was supported by the lower AIC and BIC, and by likelihood ratio comparisons. We considered a model with a linear term for age as well as a model introducing additional quadratic or inverse terms. The inclusion of either a quadratic or inverse age term improved the model by providing a better representation of the observed cyst counts at younger ages. We selected the inverse of age as it has a more straightforward biological interpretation and provides credible figures (unlike the use of a quadratic term) when extrapolating the model to pigs older than those in our experiment. Covariates such as the pig’s farm of origin, the viability of the egg pool, and the sex of the pigs were also tested, but none significantly affected the model coefficients or improved model fit. The coefficients of the final model, along with their respective confidence intervals, are presented in Table 2, while the model’s predictions, along with their credible intervals and observed values, are depicted in Fig. 2.

**Table 2:** Coefficients, standard errors, *z*-values, *P*-values, and 95% confidence intervals for the negative binomial regression model predicting the number of live cysts. The model includes the intercept, age, and the inverse of age as predictors

Variable	Estimate	Standard error	*z*-value	*P*-value	95% confidence interval	
					Minimum	Maximum
(Intercept)	10.847	1.596	6.80	< 0.001	7.36	14.30
Age	−0.269	0.074	−3.64	0.0003	−0.42	−0.10
Inverse of age	−20.335	6.585	−3.09	0.0022	−33.64	−5.51

### Serological results

All pigs used in this experiment started with no bands on LLGP-EITB and with an antigen optical density ratio < 1. Additionally, all pigs exhibited seroconversion of at least one LLGP-EITB band after infection, occurring between 3 and 9 weeks post-infection, with a median seroconversion time of 5 weeks. Following the initial seroconversion, all pigs, except for one (which had only one live cyst out of a total of two), maintained seroconversion and typically retained or increased the number of LLGP-EITB bands, which has been shown to be correlated with the presence and level of infection in prior literature [27, 28]. Seroconversion on the antigen test consistently occurred before the LLGP-EITB test, between 1 and 9 weeks post-infection, with a median time of 3 weeks. All pigs exceeded the threshold of one (meaning they were positive on the antigen test) [18] at least once after infection; in 38 pigs, the optical density ratio exceeded 3 at least at some point after infection, nine maintained values lower than 3, and five had an initial increase followed by a progressive decrease until the time of necropsy. These five pigs all had fewer than 10 live cysts. Individual serological results for each pig are presented in Supplementary Material, Fig. S1. These results, which are important to refine our understanding of how serology relates to infection, will be analyzed in detail in a future publication.

## Discussion

High variability in parasite burden is observed in animals infected with cestodes, particularly *T. solium*. Innate immunity may contribute to these effects. Statistical analysis of the results of this study demonstrated a relationship between age and cyst burden. These results suggest that the immunity of naïve pigs (not previously exposed) is greater in younger and older pigs, and lowest around 9 weeks of age, with the relationship between age and the logarithm of the mean number of cysts best represented through the sum of two terms, one proportional to age and one proportional to inverse of age.

There are many challenges in measuring the contribution of immunity to cyst development, including the effect of pig genetic lineage and variability in egg batches and numbers [13], and potentially the host’s microbiome or stress. In this study, several of these variables were controlled for, including through the use of pooled eggs from different tapeworms with confirmed viability and hatching rates, as well as direct inoculation into the stomach to ensure consistent exposure. However, genetic variability among animals could not be controlled in the design of the experiment. The pigs we used came from commercial farms where standardized purebred lines are not commonly maintained. Instead, modern commercial pig production relies on crossbred lines selected by producers to optimize productivity based on growth rate, feed efficiency, and carcass quality. The specific mixed breeds used were typical of the northern Peru region, resulting from genetic improvement programs in rural areas, and commonly found in Peruvian villages. To meet the strict age group requirements for the study, pigs were sourced from multiple granges. It is plausible that granges may differ somewhat in the genetic makeup of their pigs; hence, we included the farm of origin among the explanatory variables we considered in the statistical analysis and were able to demonstrate that inclusion of this variable did not improve the model. Note that, to our knowledge, no study has ever been undertaken regarding differences in susceptibility to infection among pig breeds or lines, so we could not rely on the literature to support or disprove a possible link between pig genetics and susceptibility.

The relatively high variability observed within age groups highlights the complexity of the factors that determine cyst development, even under controlled experimental conditions, emphasizing the importance of accounting for such heterogeneity in future investigations.

Several explanations have been put forward to justify the change in susceptibility due to age. With respect to younger ages, in rats infected with *T. taeniaeformis*, this change was attributed to little or no proteolytic activity in their intestines to assist in the hatching of eggs [29]. However, several other components could also be involved, such as innate immunity transferred by the mother, or pH or other differences in gastric fluids. We ruled out passive transfer of maternal acquired immunity because the mothers were free of infection. Evidence from other *Taenia* species also suggests that the passive transfer of maternal acquired antibodies does not play a role [12, 30].

Our research group conducted a previous study of the effect of age on infection in which pigs were infected at 1, 3, and 5 months with a single proglottid each. While the current study has coherent results for older pigs, there are differences in the youngest group: in the earlier study, pigs infected at 1 month of age had the highest mean live cyst counts and percentage of viable cysts [14]. However, this method of infection is subject to the large variability in the number of eggs per proglottid, reported to range from 3900 to 120,000 eggs [31], which is one of the main limitations we wanted to address in the current study. Given this important distinction, comparison of results across these two studies is challenging. 

The increased susceptibility observed at the natural weaning age [32, 33] and the progressive resistance to infection with age seen in this study are consistent with patterns commonly reported in other cestodes, though these trends have not been thoroughly described [12, 13]. The peak susceptibility coincides with the natural weaning age, but we can rule out weaning itself as a causal factor, as the 4-week-old pigs in this study were forcibly weaned. If immunosuppression or stress due to weaning had been significant, it would also have affected these pigs.

Regarding the increased resistance with age in older pigs, experimental infections with limited numbers of animals [34, 35], as well as evidence from mass necropsies in endemic areas, suggest the potential for complete resistance to infection over time [36, 37], even though this has not been completely demonstrated experimentally for *T. solium*. Given that the pigs had no prior exposure, as confirmed by two serological methods, this decrease in susceptibility can only be attributed to a progressive strengthening of innate immunity. Nonetheless, certain stress factors may influence susceptibility. For instance, farrowing has been reported to predispose sows to reinfection due to immunosuppressive effects during this period [38]. This phenomenon has been used to explain the presence of cysticerci with different microsatellite patterns in a naturally infected sow, suggesting infections by distinct tapeworms at different times [39, 40].

Possibly the best description of a similar pattern of infection at different ages comes from two separate studies involving *T. hydatigena* in lambs grazed on pastures permanently contaminated by experimentally infected dogs. These studies reported a progressive increase in the number of live cysts from weeks 1 to 12 post-infection [41], followed by a progressive decrease from 3 to 6 months [42]. In this case, the total number of cysts also differed by age, with younger lambs showing a lower proportion. While these findings were partly attributed to lower ingestion of contaminated pasture, they were also linked to passive immunity transferred from the mother or a combination of these factors. Comparing these results with the experimental infection reported here, it is also plausible that susceptibility to infection is influenced by the age at which exposure occurs.

Given the marked difference in the number of live cysts between ages, accompanied by an inverse pattern in degenerated cysts and a marginal difference in the number of total cysts (following the same pattern as live cysts), our results might be due to a pre-encystment immunity. This concept, introduced by Gemmell and Soulsby [43], describes the early-stage challenges faced by the oncosphere after ingestion. These challenges include exposure to gastric acid in the stomach, interaction with the intestinal epithelium, circulation through the bloodstream, and eventual implantation and growth within muscular tissue to form larvae. During the pre-encystment phase, something—either in transit or at the site of implantation—reduced their ability to develop into viable cysts. The exact mechanisms or factors responsible for this differential success rate remain unclear, highlighting a need for further research into the specific host or environmental factors affecting this process.

One of our primary aims in undertaking this study was to use the results to inform inclusion of pig immunity into our agent-based model, CystiAgent (and associated neurocysticercosis model, CystiHuman), to improve simulations of endemic transmission and the effect of interventions [44–48]. The interaction of age-related pig susceptibility and the environmental exposure to *T. solium* eggs, which eventually leads to infection, depicts a complex scenario in which multiple processes and factors likely interact across varying scales of time and space. Among the practical implications are the optimal age at which to undertake interventions in pigs (e.g., vaccination/treatment of younger vs. older pigs) and the frequency of intervention that could be most cost-effective. Further investigation is needed to explore additional factors influencing the infection patterns of *T. solium*, particularly acquired immunity.

This study has several limitations that should be considered when interpreting the findings. First, the pigs used in the experiments came from different farms, which may introduce variability in their baseline immunity, health status, or other intrinsic factors. Additionally, while our infection system ensures the controlled uptake of a precise number of eggs, it does not fully replicate natural infection dynamics. Natural infections are likely to occur gradually, with animals being exposed to varying doses of eggs over time, starting at a young age. In contrast, our experimental design involved a single high-dose exposure (20,000 eggs), which may not fully reflect field conditions and could influence the immune response observed.

Moreover, our method of delivering eggs directly into the stomach bypasses natural routes of exposure, such as oral ingestion, which could impact how the immune system is activated. While the difference between these methods may be minimal, it remains a factor to consider. Finally, as an experimental system, the controlled nature of our study does not fully replicate the complexity of real-world scenarios, such as continuous low-level exposure in highly endemic areas. Future studies should explore the effects of reinfections and different infection doses to better understand the natural immune response and its implications for disease control strategies.

## Conclusions

This study represents the first controlled infection trial in pigs using *T. solium* eggs that effectively account for key confounding factors. Our findings reveal that younger pigs exhibit partial protection against developing cysticerci compared to those infected at natural weaning age. Moreover, beyond that age, susceptibility to infection progressively decreases with age. Although our results demonstrate that higher susceptibility occurs around 7 and 16 weeks of age, the maximum susceptibility estimated by our model is likely to occur around 9 weeks of age. These results provide critical insights into the age-related dynamics of porcine susceptibility to *T. solium* and have significant implications for understanding the epidemiology and control of cysticercosis.

## Supplementary Information

Supplementary Material 1.

## Data Availability

The data collected for this study are publicly available in Mendeley Data: Gonzales-Gustavson, Eloy (2025), “Age at Infection as a Key Predictor of Cyst Burden in Pigs Experimentally Infected with *Taenia solium*,” Mendeley Data, V1, 10.17632/cdyfk73scx.1. For any additional inquiries, the corresponding author may be contacted.
