# Supplementary material for: Age at infection as a key predictor of cyst burden in pigs experimentally infected with Taenia solium
Source: Parasit Vectors. 2025 Sep 24;18:375. doi: 10.1186/s13071-025-06844-6 (PMC12462264; doi:10.1186/s13071-025-06844-6)

**Supplementary information**

Table S1. Median, minimum, and maximum weight of pigs in kilograms (kg) at the time of experimental infection, categorized by age.

|  | weight at infection (kg) | | |
| --- | --- | --- | --- |
| Age at infection | Median | Minimum | Maximum |
| 4 weeks | 7.7 | 5.7 | 12 |
| 7 weeks | 5.6 | 5 | 9.7 |
| 10 weeks | 18.3 | 9.5 | 33 |
| 13 weeks | 20 | 17 | 21 |
| 16 weeks | 16 | 12 | 37 |
| 22 weeks | 68 | 60.7 | 80 |

**Fig S1.** Individual serological response of each pig throughout the experiment. Each plot shows the weekly results of two serological tests: LLGP-EITB (bars, right Y-axis) and antigen ELISA (red line, left Y-axis). EITB responses are represented as bars with values ranging from 0 to 7, indicating the number of reactive glycoprotein bands. Antigen ELISA values are shown as a continuous red line. Time in weeks is shown on the X-axis. Each row corresponds to animals grouped by age at the time of infection: pigs infected at 4 weeks (first row), 7 weeks (second row), 10 weeks (third row), 13 weeks (fourth row), 16 weeks (fifth row), and 22 weeks of age (sixth row). The first green triangle indicates the time of experimental infection, and the second triangle indicates the time of necropsy.


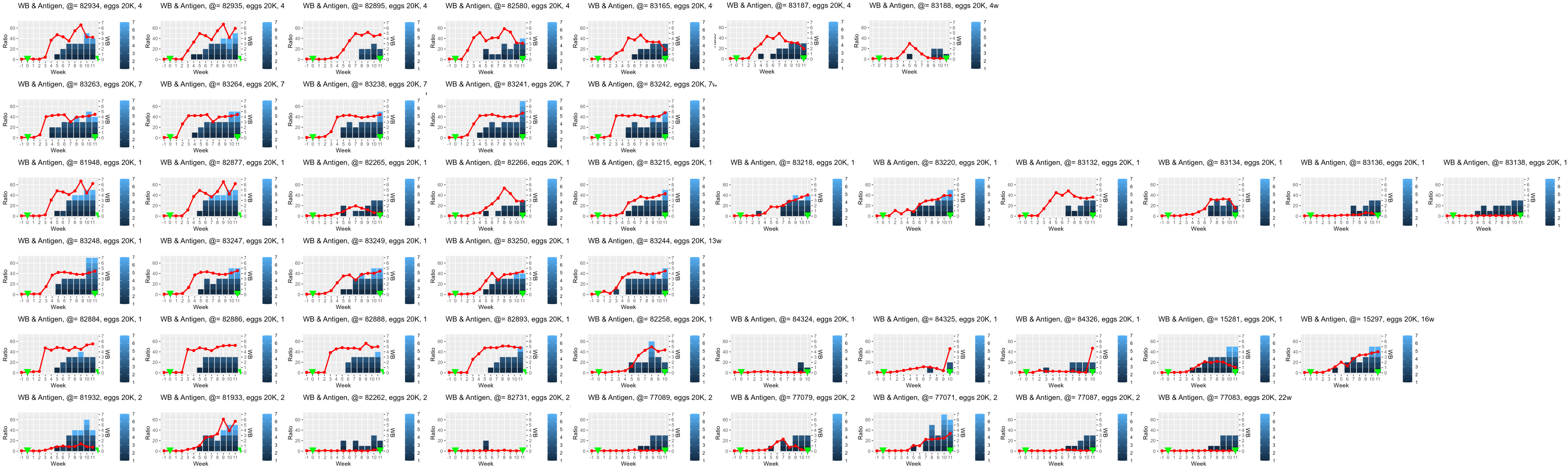

Supplement: Supplementary file 1 — Supplementary Material 1. [file 13071_2025_6844_MOESM1_ESM.docx]
